# Hospital privatization in Georgia: lessons from a radical market reform

**DOI:** 10.3325/cmj.2026.67.320

**Published:** 2026-08

**Authors:** Tengiz Verulava

**Affiliations:** Caucasus University, Tbilisi, Georgia

Georgia’s hospital privatization represents one of the most extensive market-oriented transformations undertaken in a post-Soviet health care system. Similar market-oriented reforms were introduced across several former Soviet countries, although few pursued privatization as extensively as Georgia (1). While many transition countries adopted mixed public-private arrangements, Georgia moved further by transferring most hospital ownership and service provision to private actors. Two decades later, this experience remains highly relevant as governments across Europe and other regions increasingly rely on public-private partnerships, strategic purchasing, and private investment to address fiscal constraints, infrastructure needs, and growing health care demand.

The reform process evolved through three interconnected phases. The first phase (1990s-2003) focused on restructuring the inherited Semashko system, which was characterized by excessive hospital capacity, deteriorating infrastructure, and severe fiscal constraints. Supported by international organizations, including the World Bank, reforms aimed to downsize infrastructure, decentralize management, and introduce market mechanisms (2). Following the collapse of the Semashko model, health care financing evolved through a fragmented combination of social health insurance, general taxation, and substantial out-of-pocket payments.

The second phase (2007-2010), known as the “100 Hospitals” program, marked the most intensive privatization period. Hospitals were transferred to private investors under contractual obligations to rebuild facilities and modernize infrastructure. The third phase (2010-2012) further consolidated privatization through vertically integrated insurance-provider arrangements linking financing and service delivery within private corporate structures. Although international organizations continued to provide technical assistance and policy advice, these reforms were primarily driven by domestic political and economic priorities (3).

The results were visible and immediate. Private investment rapidly modernized hospital infrastructure, replacing many obsolete Soviet-era facilities with contemporary hospitals equipped with modern technology. Managerial practices became more performance-oriented, and operational efficiency improved in several urban areas (4).

However, Georgia’s experience also revealed important policy tensions. Ownership transfer occurred more rapidly than the development of regulatory and stewardship capacity. As the state withdrew from direct ownership and provision, regulatory institutions remained comparatively weak in areas such as competition policy, quality oversight, pricing regulation, and geographic planning. Market incentives directed investment toward profitable services and densely populated urban areas, which contributed to persistent disparities in access between urban and rural regions (5). For example, hospital investment became concentrated in Tbilisi and other major urban centers, whereas many rural municipalities continued to experience shortages of specialized services despite overall infrastructure modernization. These patterns illustrate how market-based resource allocation may diverge from population health needs when regulatory and planning mechanisms remain underdeveloped.

The introduction of vertically integrated insurance-provider arrangements generated additional concerns regarding transparency, competition, and potential conflicts of interest. These developments highlighted the challenges of relying on market incentives in health care without sufficiently developed governance mechanisms. In response, the introduction of the Universal Health Care program in 2013 partially recalibrated the system by re-establishing the state as the principal health care purchaser (6). Nevertheless, service provision remained predominantly private, resulting in a hybrid health care system characterized by public financing and private delivery. While this model expanded access to publicly funded services, it also increased the importance of effective regulation, accountability mechanisms, and strategic purchasing to align private incentives with public health goals.

Why does this experience matter today? Many countries are currently expanding public-private partnerships, strategic purchasing mechanisms, and private sector participation in health care delivery. Georgia represents an informative case because it demonstrates both the opportunities and risks associated with rapid market transformation. The principal lesson is not that privatization is inherently beneficial or harmful, but that its outcomes depend heavily on governance capacity and reform sequencing. Consistent with comparative evidence from successful health system reforms, institutional capacity and effective governance are critical determinants of long-term system performance (7).

For policy-makers considering greater private sector involvement in hospital care, Georgia’s experience suggests that regulatory institutions should be strengthened before or alongside ownership transformation. Private investment can successfully address infrastructure deficits and improve efficiency, but modernization alone does not guarantee equitable access, accountability, or alignment with public health objectives. In health care systems where regulatory capacity remains underdeveloped, market reforms may generate unintended inequalities and reduce public sector leverage over service delivery.

As health systems worldwide continue to experiment with public-private arrangements, Georgia’s experience offers an important policy lesson: the state’s role does not diminish when health care ownership becomes private. On the contrary, effective stewardship, strategic purchasing, competition oversight, and equity-oriented regulation become even more important to ensure that efficiency gains translate into sustainable and equitable health outcomes. These findings suggest that privatization should be viewed not as a substitute for government responsibility but as a reform strategy whose success ultimately depends on the strength of public governance and institutional capacity.
